# Cell surface carbohydrates of symbiotic dinoflagellates and their role in the establishment of cnidarian–dinoflagellate symbiosis

**DOI:** 10.1038/s41396-021-01059-w

**Published:** 2021-07-20

**Authors:** Giada Tortorelli, Carsten Rautengarten, Antony Bacic, Gabriela Segal, Berit Ebert, Simon K. Davy, Madeleine J. H. van Oppen, Geoffrey I. McFadden

**Affiliations:** 1 School of Biosciences, The University of Melbourne, Parkville, VIC Australia; 2 Department of Animal, Plant & Soil Sciences, La Trobe Institute for Agriculture and Food, La Trobe University, Bundoora, VIC Australia; 3 Biological Optical Microscopy Platform, The University of Melbourne, Parkville, VIC Australia; 4 School of Biological Sciences, Victoria University of Wellington, Wellington, New Zealand; 5 Australian Institute of Marine Science, Townsville, QLD Australia

**Keywords:** Cellular microbiology, Molecular biology

## Abstract

Symbiodiniaceae algae are often photosymbionts of reef-building corals. The establishment of their symbiosis resembles a microbial infection where eukaryotic pattern recognition receptors (e.g. lectins) are thought to recognize a specific range of taxon-specific microbial-associated molecular patterns (e.g. glycans). The present study used the sea anemone, *Exaiptasia diaphana* and three species of Symbiodiniaceae (the homologous *Breviolum minutum*, the heterologous-compatible *Cladocopium goreaui* and the heterologous-incompatible *Fugacium kawagutii*) to compare the surface glycomes of three symbionts and explore the role of glycan–lectin interactions in host–symbiont recognition and establishment of symbiosis. We identified the nucleotide sugars of the algal cells, then examined glycans on the cell wall of the three symbiont species with monosaccharide analysis, lectin array technology and fluorescence microscopy of the algal cell decorated with fluorescently tagged lectins. Armed with this inventory of possible glycan moieties, we then assayed the ability of the three Symbiodiniaceae to colonize aposymbiotic *E. diaphana* after modifying the surface of one of the two partners. The Symbiodiniaceae cell-surface glycome varies among algal species. Trypsin treatment of the alga changed the rate of *B. minutum* and *C. goreaui* uptake, suggesting that a protein-based moiety is an essential part of compatible symbiont recognition. Our data strongly support the importance of D-galactose (in particular β-D-galactose) residues in the establishment of the cnidarian–dinoflagellate symbiosis, and we propose a potential involvement of L-fucose, D-xylose and D-galacturonic acid in the early steps of this mutualism.

## Introduction

Symbiodiniaceae are single-celled alveolate protists of the phylum Dinoflagellata. Dinoflagellates inhabit temperate and tropical areas as phototrophs, heterotrophs, parasites and symbionts, but the Symbiodiniaceae are most widely recognized as intracellular photosymbionts of reef-building corals [1]. Coral–Symbiodiniaceae symbiosis is a mutualism where the symbiont benefits from inorganic nutrients coming from host metabolism, and the coral host receives a large proportion of photosynthate produced by the dinoflagellates, thus providing the coral partner with most of its energy requirements and therefore underpinning the entire coral reef ecosystem [2].

The family Symbiodiniaceae comprises at least nine genera [1, 3]. The physiological differences among the genera—and even within genera—are enormous [4], and dramatically influence the physiology of the coral holobiont (i.e. the coral and its associated microbiota) and its ability to cope with environmental stressors [5]. Indeed, it is pivotal for the coral host to engage in symbiosis with specific symbiont types to survive [6].

The majority of coral species have a horizontal transmission mode and must acquire suitable symbionts from the surrounding environment [7, 8]. Symbiosis establishment via horizontal mechanisms involves attraction, recognition and uptake of ‘the right’ symbionts [2]. Although there is some knowledge about the processes of attraction [9, 10] and uptake [11, 12, 13], little is known on the mechanisms of symbiotic specificity and host–symbiont selection.

Inter-partner signalling molecules involved in symbiosis establishment between corals and symbiotic dinoflagellates are still being explored [14]. The initiation of this symbiosis has been compared to the recognition of a pathogen during microbial infection [15]. In this model, the molecular crosstalk between the two symbiotic partners is mediated by the specific interaction between taxon-specific microbial-associated molecular patterns (MAMPs) and pattern recognition receptors (PRRs). A widespread MAMP-PRR system is the glycan–lectin interaction that acts as a lock-and-key mechanism in both beneficial and detrimental symbioses [16], and this is proposed as a model for establishment of the cnidarian–dinoflagellate mutualism [15].

The cell surface of symbiotic dinoflagellates is populated with glycoconjugates, with some glycan motifs similar among species and others unique to each species [17]. Two types of sugar linkages, mannose-mannose and galactose-β(1-4)-N-acetylglucosamine, have been reported in the algal cell walls [18]. Conversely, mannose-binding lectin (Millectin; [19]), two N-acetyl-D-galactosamine binding proteins (Tachylectin-2-like lectin AtTL-2; [20]) and two D-galactose binding lectins, SLL-2 [21] and CecL [22], have been isolated from several coral species and shown to be involved in various phases of symbiosis establishment. The well-described N-glycan biosynthesis pathway in yeast, plants and humans [23] is also present in Symbiodiniaceae and is a key factor in cnidarian–dinoflagellate symbiosis establishment, highlighting the importance of the symbiont’s cell-surface sugar composition in this process. The majority of the N-glycan types found on the symbiont surface were mannose-rich, further supporting the role of mannose in symbiosis onset [24]. The sugars N-acetyl-D-galactosamine [20, 25], fucose [24] and α-glucose [25, 26] were also found on the symbiont cell surface. Several studies thus support the involvement of glycan–lectin interactions in the establishment of symbiosis between cnidarians and Symbiodiniaceae, but their roles require further exploration.

Here, we adopted a model organism approach to explore the molecules at the interface of the cnidarian–dinoflagellate symbiosis and investigate the hypothesis that a glycan–lectin interaction plays a role in the initiation of this relationship. The model is the sea anemone, *Exaiptasia diaphana*, which forms symbioses with members of the Symbiodiniaceae [15]. Importantly, *E. diaphana* can be freed of all symbionts by chemical bleaching, and then inoculated with new symbionts, thus providing an ideal platform to dissect host/symbiont recognition at a molecular level. We previously developed a matrix of host–symbiont compatibilities [27] with homologous-compatible (*Breviolum minutum*), heterologous-compatible (*Cladocopium goreaui*) and incompatible (*Fugacium kawagutii*) symbionts and three different anemone genotypes [28]. We hypothesized that the compatible symbionts (*B. minutum* and *C. goreaui*) have similar cell-surface structures that fit the lock-and-key mechanism of the host and are different from the ones on the cell surface of incompatible algae (*F. kawagutii*). Based on this concept, we explored and compared the composition of the three Symbiodiniaceae cell surfaces. A multi-step approach, comprising both well-established [20, 29, 30] and innovative methods [31], was adopted to profile the cell surface of these Symbiodiniaceae. In this way, we created a list of potential molecular candidates, that we then strategically altered to investigate their role(s) during the first stages of symbiosis.

## Materials and methods

### Experimental organisms

Three anemone genotypes (AIMS2, AIMS3 and AIMS4; [28]) of the cnidarian model *E. diaphana* sourced from the Great Barrier Reef (GBR) were used in this study. Polyps were kept in reconstituted seawater (RSS, [27]) in a growth chamber (LE-509, Thermoline Scientific) under constant temperature (27 °C), 12:12-h light:dark photoperiod cycle and 15-μmol photons m^−2^ s^−1^ irradiance (white + red LED lights, EDOLED), and fed with freshly hatched *Artemia* sp. nauplii ad libitum twice per week.

Three Symbiodiniaceae species were used in this work: *B. minutum* (MMSF 01, ITS2 type B1), *C. goreaui* (SCF 055-01.10, ITS2 type C1) and *F. kawagutii* (SCF 089.01, ITS2 type F1). These algae were chosen based on their different compatibilities with the anemone host [27]. The *B. minutum* culture was isolated from the *E. diaphana* anemones mentioned above [27], while *C. goreaui* and *F. kawagutii* were obtained from the Australian Institute of Marine Science (AIMS) where they were originally isolated from the GBR-sourced corals *Acrocopora tenuis* and *Pocillopora damicornis*, respectively. All cultures were maintained in 1-L Schott bottles with 0.2-μm membrane vented caps in a growth chamber (740FHC LED, HiPoint) under constant temperature (27 °C), 12:12-h light:dark photoperiod cycle, and 60-μmol photons m^−2^ s^−1^ of light.

### Nucleotide sugar and monosaccharide component analysis of Symbiodiniaceae cell walls

The nucleotide sugar and cell-wall monosaccharide compositions of *B. minutum*, *C. goreaui* and *F. kawagutii* were profiled using methods well established in higher plants [31, 32, 33]. For the nucleotide sugar analysis, Symbiodiniaceae cultures were harvested after 5 h of exposure to light. Fifty milligrams of algae per sample were resuspended in ice-cold methanol/chloroform (1:1 mixed solution), transferred into custom lysing matrix D 2-ml tubes (MP Biomedicals), ground in liquid nitrogen using a cryomill, then vortexed and placed at −20 °C for 2 h. Following the addition of 400-µL ice-cold water, the samples were centrifuged at 20,000 × *g* at 4 °C and the upper phase was transferred into 15-ml tubes placed on ice. The addition of ice-cold water and centrifugation steps were repeated two more times, and the supernatants were combined with the previously collected aqueous phase. Samples were frozen in liquid nitrogen and freeze-dried overnight. Sample purification was undertaken by solid phase extraction, then nucleotides sugars were detected and quantified by liquid chromatography tandem mass spectrometry (LC-MS/MS) using a 4,000 QTRAP LC/MS/MS system (SCIEX) equipped with a TurboIonSpray source (SCIEX) and an Agilent 1,100 Series Capillary LC System. The following nucleotide sugar standards were used in this study: UDP–α-D-xylose, UDP–β-L-arabinopyranose and UDP–α-D-galacturonic acid (Carbosource Services, Complex Carbohydrate Research Center); UDP–α-D-glucuronic acid, UDP–α-D-glucose, UDP–α-D-galactose, UDP–N-acetyl-α-D-glucosamine, UDP–N-acetyl-α-D-galactosamine, GDP–α-D-mannose, GDP–β-L-fucose, GDP-Glc (Sigma-Aldrich) and UDP–β-L-arabinofuranose (Peptides International; [32]). Data were acquired using Analyst 1.5.1 (SCIEX) and nucleotide sugars were quantified using MultiQuant 2.1 software (SCIEX) by linear regression of the peak areas [33].

To analyse the algal cell-wall monosaccharide composition, we took 300 mg of pelleted algae, and ground the cells in liquid nitrogen as described above. To obtain cell-wall alcohol insoluble residues (AIR), the homogenate was incubated in 100% EtOH at 100 °C for 30 min under constant shaking at 1,400 rpm. The AIR was then pelleted by centrifugation at 20,000 × *g* for 5 min. The pellet was washed twice in 70% EtOH and subsequently in acetone, and then left to dry overnight. Single sugar units (monosaccharides) were released by hydrolysis of AIRs with 2-N trifluoroacetic acid (TFA) and incubation at 120 °C for 1 h. The TFA solution was removed by evaporation overnight in a vacuum concentrator. The monosaccharide analysis was performed using High Performance Anion Exchange Chromatography coupled with Pulsed Amperometric Detection (HPAEC-PAD). Three replicates for each Symbiodiniaceae species were diluted and subsequently analysed on an ICS 6,000 (Dionex Corporation, Sunnyvale, CA) essentially as described in Rautengarten et al. 2014 [32], with the exception that the first isocratic elution step was performed with 4 mM to separate xylose and mannose, and while in a separate run 8 mM was used to separate N-acetyl-galactosamine and rhamnose. Standards comprised L-fucose, L-rhamnose, L-arabinose, C-galactose, D-galactose, D-xylose, D-galacturonic acid, D-glucuronic acid, N-acetylgalactosamine and N-acetylglucosamine. Both N-acetylgalactosamine and N-acetylglucosamine were TFA-treated prior to the run. A run of a standard mixture was performed with each sample set to enable sample quantitation by linear regression.

A one-way Anova (or a non-parametric Kruskal–Wallis test if normality criteria were not met) was performed to compare the relative amount of all monosaccharides found within a Symbiodiniaceae species and of each individual monosaccharide among *B. minutum*, *C. goreaui* and *F. kawagutii*. Pairwise comparisons were performed post hoc with Tukey’s tests or non-parametric Dunn test (*p* > 0.05).

### Confocal microscopy of Symbiodiniaceae cell surface

Fluorescent molecular probes conjugated with eight different lectins were used to explore their affinity for the glycoconjugates populating the cell surface of *B. minutum*, *C. goreaui* and *F. kawagutii*. Symbiodiniaceae cultures were divided into four aliquots of 1.5 × 10^6^ cells for each species and treatment. The pelleted cells were resuspended in 4% paraformaldehyde (PFA)/1× PBS, fixed for 24 h at 4 °C, washed in 1× PBS and stored at 4 °C. Three days later, each sample was washed (4,000 × *g*) three times in 1× PBS, then one of the fluorescent lectins was added to the algal solution at a final concentration of 1 mg/ml [29]. The fluorescent lectin conjugates used in this study, and their glycan affinities, are described in Table 1. After a 1-h incubation at room temperature in the dark, samples were washed three times in 1× PBS and cells resuspended in a final volume of 100 μl. Five microliters of each sample were placed in four wells of a teflon printed microscope slide (ProSciTech), and then mounted with ProLong gold antifade mountant (ThermoFisher scientific). Unstained controls were also prepared to profile the innate fluorescence of all algal strains. Samples were visualised with a Nikon A1R confocal laser scanning microscope with the NIS-Element software. The acquisition of signals from specific fluorophores was obtained by recording variable emission bandwidth with the virtual band mode. A 409-nm laser was used to detect chlorophyll autofluorescence from dinoflagellate cells, while the fluorophores Texas Red and AlexaFluor 568, 594 and 488 were specifically excited by the lasers reported in Table 1. An oil immersion objective of ×60 magnification and 1.4 numerical aperture was used to image three different sections of each well, hence 12 observations were made for each algal species and lectin treatment. The image of the algal species and lectin was unmixed against its negative control to remove any background signal caused by algal chlorophyll autofluorescence. A macro was developed to process Nd2 files using Fiji™ and obtain the mean fluorescence intensity (MFI) of the signal provided by the lectin binding to the symbiont surface. Twenty-five algal cells were randomly selected from each image, and hence 300 cells were analysed for each sample of a Symbiodiniaceae species incubated with each lectin. Data were tested for normality with the Shapiro–Wilk test and, when parametric assumptions were not met, we used the Kruskal–Wallis test to explore the fluorescence intensity profiles of different lectins binding to the surface of a Symbiodiniaceae species. Finally, the Tukey’s test was used for multiple comparisons (*p* > 0.05).

**Table 1:** Fluorescent molecular probes conjugated with lectins used in this study for analysis of the Symbiodiniaceae cell surface at the confocal microscope.

Lectin probe	Glycan affinity	Fluorophore	Laser
ConA	D-mannose; D-glucose	AlexaFluor 488	488
GS-II	Terminal, non-reducing α-/β-N-acetyl-D-glucosaminyl	AlexaFluor 594	561
LPA	N-acetylneuraminic acid; glycuronic acid	FITC	488
PhaL	N-acetyl-D-glucosamine	AlexaFluor 488	488
PNA	D-galactose	AlexaFluor 568	561
SBA	N-acetyl-D-galactosaminea	AlexaFluor 594	561
UEA	α-L-Fucose	Atto 594	561
WGA	N-acetyl-D-glucosamine	Texas Red	561

*ConA* concanavalin A, *GS-II* lectin from Griffonia simplicifolia, *LPA* lectin from Limulus polyphemus (horseshoe crab), *PhaL* lectin from Phaseolus vulgaris, *PNA* lectin from Arachis hypogaea (peanut), *SBA* lectin from Glycine max (soybean), *UEA* lectin from Ulex europaeus, *WGA* wheat germ agglutinin.

### Lectin array of Symbiodiniaceae cell-surface glycoproteins

To isolate glycoproteins from the Symbiodiniaceae cell surface, we used guanidinium chloride (GuHCl) extraction [34]. We confirmed that the extraction of cell-surface glycoproteins/proteins did not result in lysis of the algal cell by using Calcofluor-white to stain the cellulosic algal cell wall [35]. Calcofluor-stained algae were visualised with a Nikon A1R confocal laser scanning microscope with a 405-nm laser to detect stained cellulose and a 409-nm laser to detect chlorophyll autofluorescence from dinoflagellate cells.

For each algal species, an aliquot of 1 × 10^8^ cells/ml was centrifuged at 1500 × *g*, washed twice in 1x PBS, resuspended in 3 ml of 6-M GuHCl and divided into two tubes. After 4 h of incubation at RT under constant shaking at 800 rpm, cells were pelleted at 15,000 × *g* for 30 min at RT and discarded; the supernatant of the two tubes was then concentrated and combined with an Amicon Ultra MWCO 3-KDa filter, and the protein concentrations of the samples were checked with the Pierce BCA Protein Assay. Each algal species had *n* = 4 replicates. Samples were run on a Ray Biotech Lectin array 95, which has 95 different lectins (Supplementary information 1). One milligram per millilitre of glycoprotein suspension was run on the lectin array by Crux Biolabs Australia. The binding of glycoproteins to different lectins produced fluorescence intensity signals informative of a certain glycan abundance. The data were background-corrected, tested for normality and a one-way Anova used to explore the binding of a certain lectin among Symbiodiniaceae cell-surface glycoproteins. Finally, the Tukey’s test was used for multiple comparisons (*p* > 0.05), and the variation in *B. minutum*, *C. goreaui* and *F. kawagutii* cell-surface glycomes was visualised using principal component analysis (PCA).

### Cell-surface alteration and host–symbiont inoculation experiments

A total of 1053 anemones (oral disc diameter of ∼5 mm) was chemically bleached of algal symbionts as previously described [36]. After bleaching, anemones were kept in RSS and fed with brine shrimp. One week prior to inoculation with Symbiodiniaceae symbionts, feeding was discontinued, and anemones were transferred to 12-well plates such that each 12-well plate contained *n* = 3 anemone genotype replicates, organized randomly to minimize well effect. Each 12-well plate had *n* = 3 technical replicates, all altered with the same enzymatic or masking treatment and inoculated with one symbiont type.

The anemone surface lectins were masked with the sugars D-glucose, D-mannose, D-galactose, methyl-α- and β-D-galactose, L-fucose, D-xylose, D-galacturonic acid, D-glucuronic acid and L-rhamnose, all at a concentration of 10 mM (Table 2; [20]). Organisms were incubated in RSS supplemented with one of the sugars for an hour under the conditions described above, and subsequently inoculated with one of the three Symbiodiniaceae species. Control organisms did not receive any sugar prior to symbiont inoculation.

**Table 2:** Enzymes used to alter Symbiodiniaceae cell-surface molecules and carbohydrates used to mask *E. diaphana* cell-surface lectins, prior to inoculation experiments.

Organism	Treatment	Product #	Concentration	Enzyme specificity
Symbiodiniaceae	α-amylase	A6255	5 mg/ml	α-(1,4) glycan linkages
Symbiodiniaceae	Trypsin	T6567	6 μg/ml	Peptides on the C-terminal side of lysine and arginine residues
*E. diaphana*	D-galactose	G0750	10 mM	*/*
*E. diaphana*	Methyl-α-D-galactose	G1000	10 mM	*/*
*E. diaphana*	Methyl-β-D-galactose	G1125	10 mM	*/*
*E. diaphana*	D-glucose	47829	10 mM	*/*
*E. diaphana*	D-mannose	M6020	10 mM	*/*
*E. diaphana*	D-galacturonic acid	48280	10 mM	*/*
*E. diaphana*	D-glucuronic acid	G5269	10 mM	*/*
*E. diaphana*	L-fucose	F2252	10 mM	*/*
*E. diaphana*	L-rhamnose	W373011	10 mM	*/*
*E. diaphana*	D-xylose	PHR2102	10 mM	*/*

All products were purchased from Sigma-Aldrich.

The symbiont cell surface was altered via digestion with one of the two enzymes: α-amylase [29] or trypsin [18, 26, 29, 30]. Enzyme specificities are reported in Table 2. Algal cells were sampled in the exponential growth phase, pelleted and resuspended in a digestion solution composed of RSS and one of the enzymes used in the study. After a 2-h digestion step at 27 °C in the dark in a shaking incubator at 90 rpm, the symbionts were washed twice in RSS and used immediately to inoculate the bleached, unmodified anemones. Control organisms received untreated symbionts.

For both host and symbiont surface modification experiments, inoculations were performed as previously described [27]. Given that most Symbiodiniaceae cell-surface glycans recover within 48–72 h post-enzymatic cleaving [30], anemones were anesthetized with MgCl_2_ 2 days post-inoculation, and tentacles then sampled, fixed and microscopically analysed to assess symbiont cell densities in *E. diaphana* [27]. Data were log-transformed to achieve normality and a generalized linear model (GLM) with a Gaussian distribution was used to test the influence of *E. diaphana* genotype (i.e. AIMS2, 3 and 4), Symbiodiniaceae species (i.e. *B. minutum*, *C. goreaui* and *F. kawagutii*) and treatment (i.e. enzymatic treatment of symbiont surface or masking of host lectins with carbohydrates) on symbiont uptake by the host (as number of symbiont cells/mm^2^ in anemone tentacles). Analyses were conducted in R v. 3.6.1 [37] with the package Tidyverse [38]. The GLM specification for the model was:

$$	{{{\boldsymbol{glm}}}}({{{\boldsymbol{N}}}} \sim {{{{{\bf{host}}}}}}\;{{{{{\bf{genotype}}}}}} \ast {{{{{\bf{symbiont}}}}}}\;{{{{{\bf{species}}}}}} \ast {{{{{\bf{treatment}}}}}},\;{{{{{\bf{family}}}}}} = \\ 	{{{{{\bf{gaussian}}}}}}\left( {{{{{{\bf{link}}}}}} = {{{\mathrm{log}}}}} \right))$$

Best model selection was performed by comparing the full model with all the effects and interactions against the model without each of the effects or interactions and confirmed using the Akaike Information Criterion (AIC, 15395.97). Analysis of variance was used to test the significance of the overall fixed effects fitted in the model.

## Results

### Nucleotide sugar and monosaccharide composition analysis of Symbiodiniaceae cell walls

**Fig. 1:**
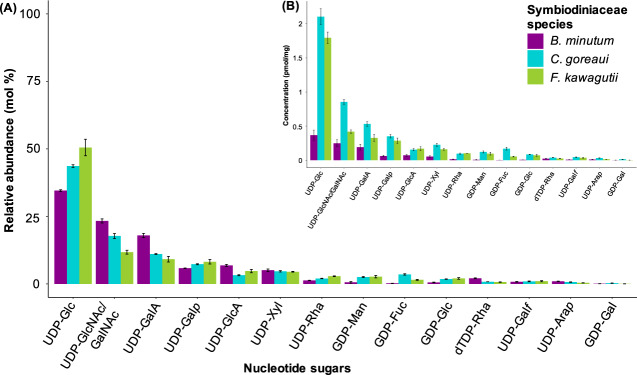
Nucleotide sugars present in *B. minutum* (purple), *C. goreaui* (blue) and *F. kawagutii* (green). **A** Each bar represents the relative abundance (mean molar percentage ± SEM) of a nucleotide sugar in the algal cell. **B** Concentration (pmol/mg ± SEM) of a nucleotide sugar in the algal cell. UDP-Glc = UDP-glucose, UDP-GlcNAc/GalNAc = UDP–N-acetylglucosamine and UDP–N-acetylgalactosamine, UDP-GalA = UDP-D-galacturonic acid, UDP-Gal = UDP-galactose, PAPS = adenosine 3′-phosphate 5′-phosphosulfate, UDP-Xyl = UDP-xylose, UDP-GlcA = UDP-D-glucuronic acid, GDP-Fuc = GDP-fucose, GDP-Man = GDP-mannose, UDP-Rha = UDP-rhamnose, GDP-Glc = GDP-D-glucose, UDP-Galf = UDP-galactofuranose, dTDP-Rha = dTDP-rhamnose, UDP-Arap = UDP-arabinopyranose, GDP-Gal = GDP-galactose.

The surface of the symbiont cell constitutes an intricate mesh of cellulose, glycoproteins and polysaccharides [17]. In eukaryotes, matrix polysaccharides are synthesized from activated sugar substrates called nucleotide sugars, most of which are transferred by nucleotide sugar transporters from the cytosol into the Golgi apparatus where they are assembled into glycan polymers by various glycosyltransferases, and then transported via vesicles to the cell wall [31]. Therefore, we decided to investigate the nucleotide sugar profiles from whole cells to gain insight into possible end products present in the dinoflagellate wall. We detected various nucleotide sugars in the three species of Symbiodiniaceae investigated (Fig. 1). The respective sugar residues can be present in cell-wall polymers, glycoproteins and/or surface glycans.

**Fig. 2:**
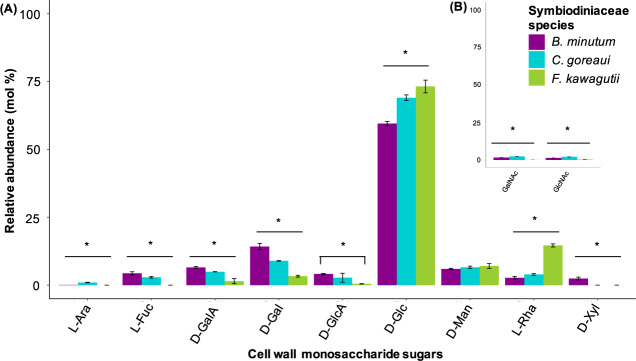
Monosaccharide composition of hydrolysed alcohol insoluble residues extracted from the cell wall of *B. minutum* (purple), *C. goreaui* (blue) and *F. kawagutii* (green). **A** Monosaccharide analysis first isocratic elution step was performed with 4 mM or **B** with 8 mM. Each bar represents the relative abundance (mean molar percentage ± SEM) of a monosaccharide. Stars indicate statistically supported differences. L-Ara = L-arabinose, L-Fuc = L-fucose, D-GalA = D-galacturonic acid, D-Gal = D-galactose, D-GlcA = D-glucuronic acid, D-Glc = D-glucose, D-Man = D-mannose, L-Rha = L-rhamnose, D-Xyl = D-xylose, GalNAc = N-acetylgalactosamine, GlcNAc = N-acetylglucosamine.

We then examined the monosaccharide composition of insoluble cell-wall extracts from each of the three Symbiodiniaceae species (Fig. 2). All cell-wall monosaccharides identified were also evident in their respective precursor form in the nucleotide sugar analysis (Fig. 1). The most abundant monosaccharide detected in the three species was glucose (Fig. 2). D-mannose was the only monosaccharide for which the relative amount did not statistically differ among the three Symbiodiniaceae species (*p* > 0.05; Fig. 2). The relative amounts of L-fucose, L-rhamnose, D-galactose, D-glucose, D-xylose and D-galacturonic acid differed among species (*p* < 0.05; Fig. 2), while D-glucuronic acid was dissimilar between *B. minutum* and *F. kawagutii* only (*p* < 0.05; Fig. 2). L-arabinose, N-acetyl-galactosamine and -glucosamine were present in trace amounts. We therefore did not further investigate the effect of these monosaccharides on symbiosis establishment.

### Confocal microscopy of Symbiodiniaceae cell surfaces

**Fig. 3:**
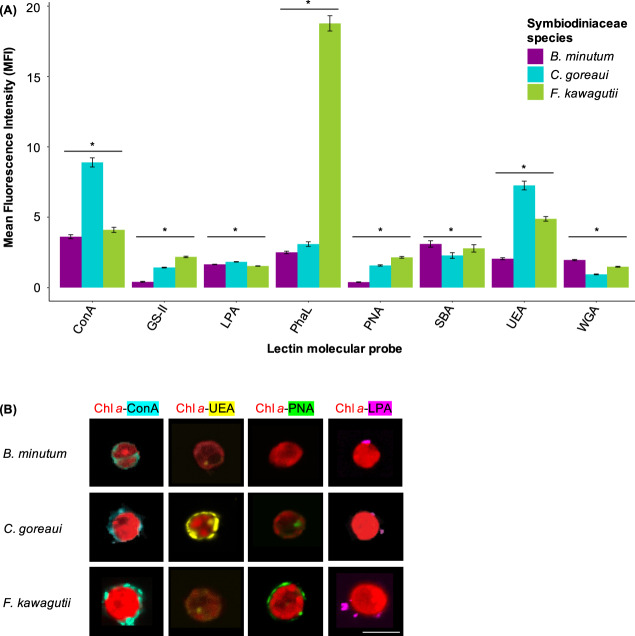
Confocal microscopy of  *B. minutum* (purple), *C. goreaui* (blue) and *F. kawagutii* (green) cell surfaces. **A** Mean fluorescence intensity (MFI ± SEM) of lectin conjugate binding to the cell surface of a Symbiodiniaceae species. ConA = concanavalin A, GS-II = lectin from *Griffonia simplicifolia*, LPA = lectin from *Limulus polyphemus* (horseshoe crab), PhaL = lectin from *Phaseolus vulgaris*, PNA = lectin from *Arachis hypogaea* (peanut), SBA = lectin from *Glycine max* (soybean), UEA = lectin from *Ulex europaeus*, WGA = wheat germ agglutinin. Stars indicate statistically supported differences. **B** Representative cells of *B. minutum*, *C. goreaui* and *F. kawagutii* labelled with the lectin probes ConA (concavalin A specific for D-mannose and D-glucose; light blue), UEA (lectin from *Ulex europaeus* specific for α-L-fucose; yellow), PNA (lectin from *Arachis hypogaea* specific for lactose and D-galactose; green) and LPA (lectin from *Limulus polyphemus* specific for N-acetylneuraminic acid and glucuronic acid; magenta) and imaged using confocal microscopy. Scale = 10 μm.

The MFI profile of different lectin conjugates varied among the three Symbiodiniaceae species (Fig. 3). The lectins ConA, GS-II, LPA, PhaL, SBA, UEA and WGA bound significantly to the surface of *B. minutum*. The *C. goreaui* surface was populated by glycans with an affinity to ConA, GS-II, LPA, PhaL, PNA, SBA and UEA. The surface of *F. kawagutii* exhibited significant binding for all the lectin conjugates used in the study. ConA, which specifically binds to terminal D-mannose and D-glucose residues, had a stronger intensity profile in *C. goreaui* compared with *B. minutum* and *F. kawagutii* (*p* < 0.001). The latter two species showed similar binding of the probe to their cell surfaces. The lectins GS-II, LPA, PhaL, SBA and UEA all bound to *B. minutum*, *C. goreaui* and *F. kawagutii*, and showed different MIFs among symbiont species. *C. goreaui* and *F. kawagutii* presented different amounts of D-galactose on their cell surfaces, as revealed with the lectin probe PNA (*p* < 0.001); however, the lectin WGA identified a dissimilar abundance of glycoconjugates containing N-acetyl-D-glucosamine residues on *B. minutum* and *F. kawagutii* (*p* < 0.001).

### Lectin array of Symbiodiniaceae cell-surface glycoproteins

Confocal microscopy showed an intact Calcofluor-stained cell wall surrounding the Symbiodiniaceae cells after the GuHCl treatment (Supplementary information 2), which we interpret as the extraction procedure not disrupting the majority of cells.

**Fig. 4:**
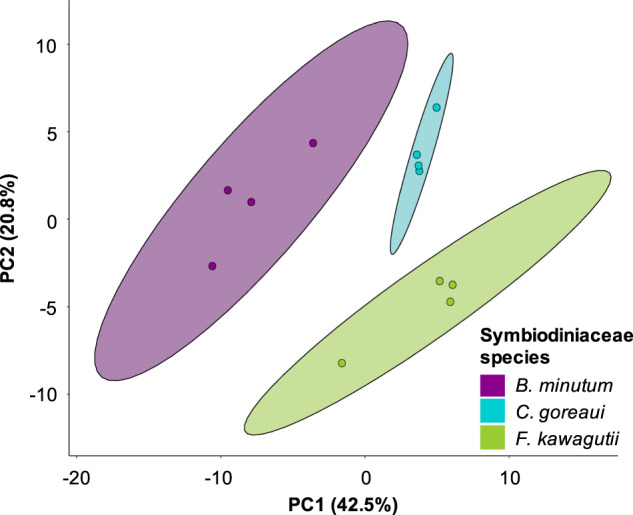
Affinity of *B. minutum* (purple), *C. goreaui* (blue) and *F. kawagutii* (green) cell-surface glycoproteins for lectins. PCA visualization of Symbiodiniaceae glycoconjugates binding to 95 lectins on the array.

Fifty-four percent of the lectins showed a difference in their affinity to the three Symbiodiniaceae species (Supplementary information 1 and 3). In line with the confocal microscopy results, the Symbiodiniaceae surface glycome varied among *B. minutum*, *C. goreaui* and *F. kawagutii*, and PCA showed that cell-surface glycoproteins clustered according to symbiont species (Fig. 4). The presence/absence of D-mannose, D-glucose, D-galactose, L-fucose, N-acetyl-D-galactosamine and N-acetyl-D-glucosamine identified via confocal microscopy was confirmed by the lectin array, but the array did not detect any statistically supported difference among algal species for the lectins PhaL, SBA, UEA and WGA (Supplementary information 1 and 3). This discrepancy may be attributed to the sensitivity of each method, as we discuss later in this work.

Lectins of the array that were specific for NANA showed a light binding to the glycoproteins of the three algal cells (Supplementary information 3), as also shown by the binding of the LPA lectin probe (Fig. 3). Sialic acids, usually characteristics of higher invertebrates, have been found composing a small percentage of the Symbiodiniaceae N-glycome [24]. Future directions should explore the role of these molecules in the algal cell.

### Cell-surface alteration and host–symbiont inoculation experiments

**Fig. 5:**
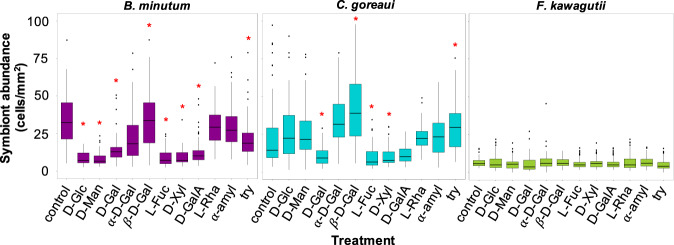
Cell surface alterations and inoculation experiments of the host with *B. minutum* (purple), *C. goreaui* (blue) and *F. kawagutii* (green). Symbiont abundance (cells/mm^2^) in the tentacles of *E. diaphana*, 48 h post-inoculation after masking of the host surface lectins with the carbohydrates D-Glu = D-glucose, D-Man = D-mannose, D-Gal = D-galactose, α-D-Gal = methyl-alpha-D-galactose, β-D-Gal = methyl-beta-D-galactose, L-Fuc = L-fucose, D-Xyl = D-xylose, D-GalA = D-galacturonic acid, L-Rha = L-rhamnose or enzymatic treatment of the Symbiodiniaceae surface with α-amyl = α-amylase or try = trypsin, and compared to the control. Red stars indicate statistically supported differences compared to control.

The density of *B. minutum* and *C. goreaui* populating *E. diaphana* at 48 hpi varied considerably (Fig. 5 and Table 3). Congruent with findings in our previous study [27], *E. diaphana* genotype influenced the start of symbiosis (GLM, df = 2, *p* < 0.001; Supplementary information 4), although a clear pattern between host identity and symbiosis establishment was not found. Symbiont species (GLM, df = 1, *p* < 0.001), treatment (GLM, df = 12, *p* < 0.001) and the interaction of the three factors (GLM, df = 24, *p* < 0.001) significantly affected the establishment of new associations. The homologous species (*B. minutum*) colonised the anemones rapidly, whereas the compatible heterologous species (*C. goreaui*) also colonised the animals, but at a lower density, and the incompatible strain (*F. kawagutii*) did not measurably colonise the hosts (Fig. 5 and Table 3).

**Table 3:** Effect of treatments (host lectin masking and symbiont cell-surface enzymatic modification) on the colonization of *E. diaphana* by *B. minutum*, *C. goreaui* and *F. kawagutii*.

Treatment	Symbiodiniaceae species	≠compared to control	Effect on colonization
D-glucose	*B minutum*	***	↓
	*C. goreaui*	.	−
	*F. kawagutii*	.	−
D-mannose	*B minutum*	***	↓
	*C. goreaui*	.	−
	*F. kawagutii*	.	−
D-galactose	*B minutum*	***	↓
	*C. goreaui*	*	↓
	*F. kawagutii*	.	−
α-D-galactose	*B minutum*	.	−
	*C. goreaui*	.	−
	*F. kawagutii*	.	−
β-D-galactose	*B minutum*	*	↓
	*C. goreaui*	***	↑
	*F. kawagutii*		−
L-fucose	*B minutum*	***	↓
	*C. goreaui*	*	↓
	*F. kawagutii*	.	−
D-xylose	*B minutum*	***	↓
	*C. goreaui*	*	↓
	*F. kawagutii*	.	−
D-galacturonic acid	*B minutum*	**	↓
	*C. goreaui*	.	−
	*F. kawagutii*	.	−
L-rhamnose	*B minutum*	.	−
	*C. goreaui*	.	−
	*F. kawagutii*	.	−
α-amylase	*B minutum*	.	−
	*C. goreaui*	.	−
	*F. kawagutii*	.	−
Trypsin	*B minutum*	*	↓
	*C. goreaui*	*	↑
	*F. kawagutii*	.	–

Stars indicate statistically supported differences of the number of cells/mm^2^ of a Symbiodiniaceae species compared to the control. . = *p* > 0.05. (↓) means the treatment decreased re-infection of the anemones by the symbiont; (–) means the treatment had no influence and allowed re-infection of the anemones by the symbiont; (↑) means the treatment resulted in a super-infection of the anemones by the symbiont.

*p < 0.05; **p < 0.01; ***p < 0.001.

After exposing aposymbiotic anemones to certain monosaccharides, we observed significantly lower in hospite *B. minutum* and *C. goreaui* cell densities at 48 hpi for D-galactose (*p*_*B*_ < 0.001; *p*_*C*_ < 0.05), L-fucose and D-xylose (*p*_*B*_ < 0.001; *p*_*C*_ < 0.05). Incubation of the aposymbiotic hosts with D-glucose, D-mannose or D-galacturonic acid resulted in lower algal densities (compared to the controls) for *B. minutum,* but revealed no difference for *C. goreaui* (*p*_*B*_ < 0.001; *p*_*C*_ > 0.05). Exposing the hosts to α-D-galactose and L-rhamnose had no effect on symbiosis establishment with any of the two compatible symbionts (*p* > 0.05), whereas the presence of β-D-galactose resulted in a super-infection for both *B. minutum* and *C. goreaui* (*p* < 0.001). The inability of *F. kawagutii* to colonise *E. diaphana* did not change (*p* > 0.05) as a result of any of the monosaccharide host-masking trials (Fig. 5 and Table 3). Incubation of *E. diaphana* with D-glucuronic acid was lethal to the anemones, hence it was not possible to collect data for this treatment.

Digesting proteins at the symbiont surface with the enzyme trypsin prior to inoculation significantly affected the uptake of both compatible symbionts (*p*_*B*_ < 0.001; *p*_*C*_ < 0.05), severely reducing the success of *B. minutum* but marginally increasing *C. goreaui* cell density (Fig. 5 and Table 3). Alpha-amylase had no influence on the density of either *B. minutum* or *C. goreaui* found in the host compared to the control (*p* > 0.05; Fig. 5 and Table 3). The infectivity of *F. kawagutii* for all genotypes of *E. diaphana* did not differ significantly (*p* > 0.05) from the control after α-amylase or trypsin pre-digestion (Fig. 5 and Table 3).

## Discussion

We explored the involvement of glycan–lectin interactions in the establishment of endosymbiosis between the sea anemone, *E. diaphana* and three species of Symbiodiniaceae algae, the homologous *B. minutum*, the heterologous-compatible *C. goreaui* and the heterologous-incompatible *F. kawagutii*.

As expected, we found good concordance between the nucleotide sugar repertoire in the three Symbiodiniaceae species and our monosaccharide analysis, which is consistent with these precursors finding their way into glycan structures such as those making up the cell walls. The cell-surface glycome varies among *B. minutum*, *C. goreaui* and *F. kawagutii*, as shown by the monosaccharide analysis (Fig. 2), confocal microscopy (Fig. 3) and the lectin array results (Fig. 4 and Supplementary information 3).

### Glucose and mannose

D-glucose stands out as highly abundant in both the nucleotide sugar pools and in the cell-wall extracts of the three Symbiodiniaceae species (Figs. 1 and 2). The symbiont cell wall is predominantly composed of cellulose [17], a polysaccharide consisting of β-linked D-glucose, so the abundance of this molecule across the three algal species is not surprising. Binding of the lectin molecular probe ConA, which is specific for D-glucose (and D-mannose), to all the three algal species (Fig. 3) is also consistent with cellulose in the algal cell walls. *B. minutum*, *C. goreaui* and *F. kawagutii* cell-surface glycoproteins bound to several lectins specific for glucose and mannose in the lectin array study (i.e. LcHA and PSA; Supplementary information 1 and 3). When we treated the anemones with D-glucose prior to inoculation with symbionts, it significantly decreased the colonisation success of the homologous *B. minutum* but had no impact on the colonisation success of *C. goreaui* and *F. kawagutii* (Fig. 5 and Table 3).

D-mannose was the only sugar that did not differ significantly in mole percentage amongst the three species of Symbiodiniaceae (Fig. 2). Previous work has shown that high-mannose glycans constitute 52% of the *B. minutum* cell-surface N-glycome [24], and the most abundant protein on the cell surface of symbiotic dinoflagellates found by Lin and co-workers bears a terminal mannose residue [18]. Here, lectins specific for high mannose such as BC2L-A, CALSEPA, GRFT and ORYSATA recognised glycoproteins of the three Symbiodiniaceae species in the array experiment (Supplementary information 1 and 3). Furthermore, two mannose-binding lectins, ‘Millectin’ from *Acropora millepora* [19] and ‘PdC lectin’ from *Pocillopora damicornis* [39], bind around the algae in the gastrodermis of the host [39] and are characterised by extensive sequence variation of the binding region, which may reflect the capacity to recognise both beneficial symbionts and pathogens [19]. Here, treating the anemones with D-mannose prior to inoculation with symbionts decreased the colonisation success of the homologous *B. minutum* but had no effect on experiments with *C. goreaui* and *F. kawagutii* (Fig. 5 and Table 3). ConA negatively influenced symbiosis establishment between the coral *Fungia scutaria* and its symbionts [26], but, in the case of *A. tenuis*, the lectin had no effect on symbiotic colonisation [29]. Taken together, these results suggest that both D-mannose and D-glucose residues are likely important functional components of the symbiont surface that participate in recognition of Symbiodiniaceae and maintenance of the mutualism, rather than just being crucial monosaccharides responsible for the discrimination between compatible and incompatible symbionts.

### D-galacturonic acid

The relative amount of D-galacturonic acid was different among Symbiodiniaceae species, being more abundant in the compatibles *B. minutum* and *C. goreaui* compared to the non-compatible *F. kawagutii* (Fig. 2). When we incubated the anemones in D-galacturonic acid, colonisation success remained unaltered for *F. kawagutii* and decreased for both *B. minutum* and *C. goreaui*, although the decrease was only statistically supported for *B. minutum* (Fig. 5 and Table 3). We speculate that D-galacturonic acid may be a component of the glycoproteins involved in the recognition of the compatible symbionts.

### D-galactose

The presence of D-galactose molecules on the symbiont cell wall (Fig. 2) was confirmed via confocal microscopy with the lectin probe PNA (Fig. 3), and with the binding of algal surface glycoproteins to galactose-specific lectins on the array, such as MNAG, RCA120 and RCA60 (Supplementary information 1 and 3). Interestingly, monosaccharide analysis showed D-galactose to be more abundant in or on the cell wall of the homologous *B. minutum* compared to the heterologous *C. goreaui* and the incompatible *F. kawagutii* (Fig. 2). Previous studies reported the presence of D-galactose on the surface of several Symbiodiniaceae species [24, 25], and the colonisation of *A. tenuis* larvae during symbiosis establishment was negatively influenced by the presence of the sugar [20].

Here, D-galactose significantly decreased colonisation success of the homologous *B. minutum* and the heterologous-compatible *C. goreaui* in *E. diaphana*, compared to the control (Fig. 5 and Table 3). Thus, several lines of evidence support the involvement of D-galactose in the establishment of symbiosis between cnidarians and Symbiodiniaceae. For instance, the D-galactose-binding lectin SLL-2 has been isolated from the coral *Sinularia lochmodes*. This protein was localised surrounding the symbiont cells when resident in the gastrodermis of the host, and in vitro experiments showed the ability of SLL-2 to arrest the dinoflagellate in the typical symbiotic state, viz non-dividing and non-motile [21, 40, 41, 42]. Another lectin (CeCL) that binds to carbohydrate chains with a D-galactosyl moiety was found in *Fungia echinate*, and—similar to SLL-2—CeCL transformed Symbiodiniaceae cells from a flagellated and motile state to a coccoid and non-motile form [22].

Considering that the activity of CeCL on the symbiont cells was concentration-dependent [22], and that in our study D-galactose was found to be more abundant in the compatible symbionts than the incompatible one, this raises the question whether the relative amount of a certain glycan has an impact on symbiont selection. Another open question is whether the stereochemistry of a sugar is important in symbiont selection. To further explore the possible role of D-galactose in symbiont selection, we utilised different conformations of the molecule, namely methyl-α- or methyl-β-D-galactose, which are fixed isomers due to the methyl group. Intriguingly, the methyl-α-sugar had no effect on host colonisation, whereas methyl-β-D-galactose significantly altered the establishment of symbiosis between the host and compatible symbionts, by reducing the number of *B. minutum* but increasing the number of *C. goreaui* present in the host at 48 hpi (Fig. 5 and Table 3). We conclude that D-galactose, in particular D-galactose when it is β-linked rather than α-linked, is a crucial player of the lock-and-key mechanism responsible for recognising suitable symbionts during the establishment of symbiosis.

### Fucose and xylose

Like D-galactose, L-fucose and D-xylose are more abundant in extracts from the homologous *B. minutum* than the heterologous *C. goreaui* and *F. kawagutii* (Fig. 2), and the lectin probe UEA confirmed the presence of L-fucose exposed on the surface of symbiont cells (Fig. 3). Accordingly, PA-IIL and RSFUC, specific for fucose, bound to the surface glycome of the three algal species in the lectin array (Supplementary information 1 and 3). Both carbohydrates have been associated with Symbiodiniaceae before, as N-glycan glycosyltransferases for fucose (fucosyltransferases) and xylose (xylosyltransferases) are encoded in the algal genome [24]. Here, we show that both L-fucose and D-xylose decrease the colonisation success of *B. minutum* and *C. goreaui* in *E. diaphana* (Fig. 5 and Table 3). Although the role of these sugar residues at the initiation of symbiosis needs further exploration, these results suggest a function in the recognition of compatible algal types.

### N-acetyl hexosamine sugars

Our HPAEC-PAD analysis detected only small traces of N-acetylglucosamine and N-acetylgalactosamine among the cell-wall monosaccharides of *B. minutum*, *C. goreaui* and *F. kawagutii* (<1.5 mol%). Nevertheless, cell-surface glycoproteins of the three algal species bound several N-acetyl groups-specific lectins (i.e. GS-II, PhaL, SBA and WGA), either in the fluorescence microscopy assay or lectin array or both. Although Logan and co-workers showed identical binding of these probes to various Symbiodiniaceae species [25], our confocal microscopy study found that they bind differently to the surface of our strains of *B. minutum*, *C. goreaui* and *F. kawagutii* from the GBR (Fig. 3). In particular, the incompatible *F. kawagutii* presented high levels of N-acetyl-D-glucosamine compared to the compatibles *B. minutum* and *C. goreaui* (Fig. 3). In a previous study, two N-acetyl-D-galactosamine binding lectins were isolated from the scleractinian *A. tenuis*, and seem to be involved in the acquisition of Symbiodiniaceae of the *Symbiodinium* and *Durusdinium* genera [20]. In another study, carbohydrates with N-acetylglucosamine residues decreased the acquisition of compatible symbionts in *A. tenuis*. The N-acetyl-D-glucosamine-binding lectin ActL attracted compatible Symbiodiniaceae to the coral host, suggesting a role in chemotactic attraction prior to recognition and uptake of the symbiont to start symbiosis [43]. Perhaps, N-acetyl residues characterize symbionts incompatible with our host, *E. diaphana* from the GBR. The role of N-acetylated sugars remains to be determined, and further experiments are needed to uncover the role of these molecules in the cnidarians–Symbiodiniaceae symbiosis.

### Large and α-linked polysaccharides, and L-rhamnose

Treating the three symbiont species with α-amylase or challenging them against *E. diaphana* in the presence of L-rhamnose, did not alter the ability of the symbionts to colonize *E. diaphana* (Fig. 5 and Table 3). Starch-like structures are not typical components of dinoflagellate cell walls, so it is not surprising that α-amylase treatment had no effect on establishment of symbiosis here and in a previous study [26]. Indeed, α-linked polysaccharides and L-rhamnose do not seem to be crucial in establishment of the cnidarian–dinoflagellate symbiosis.

### Trypsin treatment

Trypsin is a protease that cleaves peptide bonds on the C-terminal side of lysine and arginine amino acid residues. It has been used widely on Symbiodiniaceae [18, 26, 30] to examine the contribution of symbiont cell wall or surface protein moieties to host cell invasion. Previous studies have shown that homologous symbiont colonisation success was negatively affected by trypsin treatment in *E. diaphana* from Taiwan [18] and larvae of the coral *F. scutaria* [26]. Conversely, an experiment on *A. tenuis* larvae in which glycoconjugates of algal surface glycoproteins were digested with trypsin resulted in a super-infection by *C. goreaui*, one of the compatible symbionts during the larval stage of this coral species [29]. In our study, trypsin treatment negatively influenced colonisation by the homologous *B. minutum* but enhanced uptake of *C. goreaui*, resulting in a super-infection of the anemones (Fig. 5 and Table 3). A specific configuration of glycoproteins might, on one hand, ensure the recognition of *C. goreaui* as a compatible partner; on the other hand, it might be the crucial factor that regulates the uptake of a symbiont that, although compatible, is not the homologous type of *E. diaphana* from the GBR. The alteration of these glycan structures, besides altering the establishment of symbiosis with compatible symbionts, might also allow *C. goreaui* to more easily invade the host by avoiding the host mechanism of minimizing the uptake of heterologous algae.

### Concordances and differences between the glycome analytical methods

Our multi-technology approach to characterize the glycomes of differently compatible symbionts is the first of its kind. A high degree of concordance between the results obtained from HPAEC-PAD, confocal and lectin array analyses emphasizes the utility of using multiple assays to explore the glycomes. Nevertheless, some inconsistencies between the analyses were evident. The three methods rely on very different methodologies. For the HPAEC-PAD analysis, the Symbiodiniaceae cells were lysed, AIR was extracted, hydrolysed, concentrated and analysed. For confocal microscopy, the Symbiodiniaceae cells were fixed, incubated with labelled lectins, which were then visualized by fluorescence microscopy. For lectin array analysis, the Symbiodiniaceae cell-surface glycoproteins were extracted, concentrated, purified, biotin labelled and then exposed to the array of immobilized lectins. Thus, three of the methods aim to extract the glycans and then modify them for identification by different means whereas the microscopy approach sends lectins into the cell surface hoping that the glycan target is accessible. It is not surprising that some inconsistencies between sugars identified are apparent, but the overall concordance gives confidence in these approaches. Furthermore, our lectin array—in which we used GuHCl extraction to release glycoproteins—showed marked differences among different symbiont species; differences not reported in a previous lectin array analysis using a different extraction method [30]. Detailed inventories of the surface glycomes of compatible/incompatible symbionts will help unravel which glycans are crucial to successful symbiosis.

## Conclusion

The present study explored glycan–lectin interactions during the establishment of symbiosis between cnidarians and three species of Symbiodiniaceae, each with a different compatibility with the model symbiotic cnidarian *E. diaphana*. We adopted a multi-step approach and profiled cell-surface molecules via nucleotide sugar analysis, monosaccharide composition analysis, confocal microscopy and lectin array analysis. Once we had assessed the differences between the three cell-surface glycomes, we examined the importance of selected sugar residues in the initiation of a mutualistic relationship between the three algae and three genotypes of aposymbiotic *E. diaphana* from the GBR. D-glucose and D-mannose were the most abundant monosaccharides of *B. minutum*, *C. goreaui* and *F. kawagutii*. For these sugars, we envision a role as pivotal functional components of the symbiont surface glycome, which are probably involved in the recognition between beneficial or detrimental symbionts but not necessarily associated with host discrimination among Symbiodiniaceae species. Masking of host cell-surface lectins with L-rhamnose and the alteration of the symbiont surface with α-amylase had no influence on the establishment of symbiosis, and we therefore assume that these sugar residues are not critical. Sialic acids and N-acetylgalactosamine showed an unclear pattern in the present analysis, which deserves further attention. Treatment of the algal cells with trypsin changed their rate of uptake, reinforcing previous conclusions that a protein-based moiety, likely a glycoprotein, is an essential part of compatible symbiont recognition. Intriguingly, our data also strongly support the importance of D-galactose and, in particular, β-D-galactose residues in the establishment of the cnidarian–dinoflagellate symbiosis, and we propose a potential involvement of other sugar residues such as L-fucose, D-xylose and D-galacturonic acid in the early steps of this mutualism.

## Supplementary information

Supplementary information 1, 3

Supplementary information 2, 4
