# Supplementary material for: Cell surface carbohydrates of symbiotic dinoflagellates and their role in the establishment of cnidarian–dinoflagellate symbiosis
Source: ISME J. 2021 Jul 20;16(1):190–9. doi: 10.1038/s41396-021-01059-w (PMC8290866; doi:10.1038/s41396-021-01059-w)
Supplement: Supplementary file 2 — Supplementary information 2, 4 [file 41396_2021_1059_MOESM2_ESM.docx]

**Supplementary Information 2.** Calcofluor staining of the cell wall of Symbiodiniaceae in unprocessed (a, control) cells and after GuHCl treatment (b). Cellulosic cell wall is stained in blue, and the innate chlorophyll a fluorescence of Symbiodiniaceae is in red. Scale 10 μm.

 **Supplementary Information 4.** Symbiont abundance (cells/mm^2^) for *B. minutum* (purple), *C. goreaui* (blue) and *F. kawagutii* (green), in the tentacles of three *E. diaphana* genotypes (AIMS2, 3 and 4), 48 hours post inoculation after masking of the host surface lectins with the carbohydrates D-glucose, D-mannose, D-galactose, methyl-alpha-D-galactose, methyl-beta-D-galactose, L-fucose, D-xylose, D-galacturonic acid, L-rhamnose or enzymatic treatment of the Symbiodiniaceae surface with alpha-amylase or trypsin, and compared to the control.
